# Predictive factors for breast cancer in patients diagnosed atypical ductal hyperplasia at core needle biopsy

**DOI:** 10.1186/1477-7819-7-77

**Published:** 2009-10-23

**Authors:** Byung Joo Chae, Ahwon Lee, Byung Joo Song, Sang Seol Jung

**Affiliations:** 1 Department of Surgery, Catholic University of Korea, Seoul, Korea; 2 Department of Pathology, Catholic University of Korea, Seoul, Korea; 3 Breast Center Multidisciplinary Team, Department of Surgery, Seoul St Mary's Hospital, Seoul, Korea

## Abstract

**Background:**

Percutaneous core needle biopsy (CNB) is considered to be the standard technique for histological diagnosis of breast lesions. But, it is less reliable for diagnosing atypical ductal hyperplasia (ADH). The purpose of the present study was to predict, based on clinical and radiological findings, which cases of ADH diagnosed by CNB would be more likely to be associated with a more advanced lesion on subsequent surgical excision.

**Methods:**

Between February 2002 and December 2007, consecutive ultrasound-guided CNBs were performed on suspicious breast lesions at Seoul St. Mary's Hospital. A total of 69 CNBs led to a diagnosis of ADH, and 45 patients underwent follow-up surgical excision. We reviewed the medical records and analyses retrospectively.

**Results:**

Sixty-nine patients were diagnosed with ADH at CNB. Of these patients, 45 underwent surgical excision and 10 (22.2%) were subsequently diagnosed with a malignancy (ductal carcinoma *in situ*, n = 8; invasive cancer, n = 2). Univariate analysis revealed age (≥ 50-years) at the time of core needle biopsy (p = 0.006), size (> 10 mm) on imaging (p = 0.033), and combined mass with microcalcification on sonography (p = 0.029) to be associated with underestimation. When those three factors were included in multivariate analysis, only age (p = 0.035, HR 6.201, 95% CI 1.135-33.891) was an independent predictor of malignancy.

**Conclusion:**

Age (≥ 50) at the time of biopsy is an independent predictive factor for breast cancer at surgical excision in patients with diagnosed ADH at CNB. For patients diagnosed with ADH at CNB, only complete surgical excision is the suitable treatment option, because we could not find any combination of factors that can safely predict the absence of DCIS or invasive cancer in a case of ADH.

## Background

Percutaneous core needle biopsy (CNB) is the standard technique for histological diagnosis of breast lesions. It has become the procedure of choice to investigate suspicious lesions of the breast and has been shown to be an effective means to rule out cancer, alleviating the cost and discomfort of surgery. Overall, CNB histological findings are in agreement with surgical biopsy in more than 95% of the cases [1-3]. But, CNB is less reliable for diagnosing atypical ductal hyperplasia (ADH).

ADH is a proliferative lesion of the breast epithelium, which fulfils some but not all the criteria of low grade ductal carcinoma *in situ *(DCIS) [4]. ADH carries a 4-5 times increased risk of subsequent development of invasive carcinoma in either breast, [5,6] and there is genetic evidence in cell populations associated with cancer suggesting it may even be a direct precursor of malignancy[7]. Significant discordance has been reported isn CNB diagnosis of ADH, with 7%-87% of cases proving to be DCIS or invasive carcinoma on subsequent surgical excision [8-14]. This problem arises from the difficulty in differentiating between ADH and low grade DCIS on the small volume of tissue obtained from core biopsy [15]. In addition, foci of ADH may be present at the periphery of areas of DCIS [16] and, thus, even an unequivocal diagnosis of ADH does not preclude the presence of an adjacent and more advanced lesion. Because of this underestimation (which means presence of DCIS or invasive cancer) risk, some authors have recommended a mandatory surgical biopsy, while others have discussed options between surgery and follow-up [17]. Identification of patients with ADH diagnosed by CNB who can be spared surgical excision is an area of active investigation. However, the clinical, radiologic, and pathologic parameters on which to base this decision have not been consistently identified.

The purpose of the present study was to predict, based on clinical and radiological findings, which cases of ADH diagnosed by CNB would be more likely to be associated with a more advanced lesion on subsequent surgical excision.

## Materials and methods

Between February 2002 and December 2007, 3476 consecutive ultrasound-guided CNBs were performed on suspicious breast lesions at the Seoul St. Mary's Hospital. A total of 69 CNBs led to a diagnosis of ADH, and 45 patients underwent follow-up surgical excision. Seven patients refused surgical excision and were only followed up and 12 patients were transferred other hospital as per their request while 5 were lost to follow-up. The definition employed for "histological underestimation" was a lesion diagnosed as ADH at CNB that was revealed to harbor malignant foci at follow-up surgical excision, including DCIS and invasive cancer. All patients in this study underwent clinical and radiological examination, including mammography and ultrasound. The radiological appearance of the lesion was characterized according to the American College of Radiology Breast Imaging Reporting and Data System lexicon and the final assessment categories. All lesions were evaluated for size on imaging and presence of microcalcification. Lesion size was defined as the greatest dimension on ultrasound imaging for most patients, or mammography size for patients with microcalcification dominant lesions. Ultrasound-guided biopsies were used for sonographically visible lesions, and were performed with patients in a supine or decubitus position using high-resolution sonography. The biopsy was performed using a device with a 14-gauge automated needle or with an 11-gauge vacuum assisted biopsy device. The core biopsy tissue sections were fixed in 10% formaldehyde and embedded in paraffin. Each biopsy specimen was stained with hematoxylin and eosin. The biopsy slides were reviewed by experienced pathologists and diagnosed according to the ADH diagnostic criteria of the World Health Organization guidelines. The data were analyzed using Chi-square and logistic regression, as well as Fisher exact test for the small sample. *P *values < 0.05 were considered statistically significant.

## Results

Sixty-nine patients were diagnosed with ADH at CNB. Of these, 45 underwent surgical excision at our institution. Of the 45 patients, 10 (22.2%) were diagnosed with a malignancy after surgical excision (DCIS, n = 8; invasive cancer, n = 2). Table 1 summarizes the underestimation rates and distribution in all patients according to clinical, radiological, and pathological variables, and compares the accurate diagnoses (n = 35) and underestimations (n = 10) according to patient, lesion, and biopsy variables. Six (13.3%) underwent 11-gauge stereotactic vacuum assisted biopsy because lesions were seen mainly by mammography rather than ultrasound. Women in the accurate diagnosis group were younger than those in the underestimation group (p = 0.003). Nine of the 39 ADH lesions (23.1%) found with 14-gauge automated gun biopsies were upgraded to carcinoma, and one of the six ADH lesions (16.7%) found with 11-gauge vacuum-assisted biopsies were upgraded to carcinoma. The underestimation rate for the 11-gauge vacuum-assisted biopsy (16.7%) was not significantly lower than that for the 14-gauge automated gun biopsy (23.1%) (p = 0.725).

**Table 1:** Pathologic results after surgical excision according to clinical, radiological and histological variables.

		**Pathology after excision**		**Underestimation** **rate (22.2%)**	**P value** **(Chi-square)**
				
		**Benign (n = 35)**	**Malignancy (n = 10)**		
Age (years)	< 50	28 (80%)	3 (30%)	9.7%	***0.003***
	≥50	7 (20%)	7 (70%)	50%	
					
Mass on MMG	Yes	10 (32.3%)	4 (50%)	28.6%	0.351
	No	21 (67.7%)	4 (50%)	16%	
					
MIC on MMG	Yes	8 (25.8%)	4 (50%)	33.3%	0.186
	No	23 (74.2%)	4 (50%)	14.8%	
					
Mass + MIC on MMG	Yes	1 (3.1%)	2 (22.2%)	66.7%	0.52
	No	31 (96.9%)	7 (77.8%)	18.4%	
					
Lesion size	≤1 cm	23 (69.7%)	3 (30%)	11.5%	***0.024***
	> 1 cm	10 (30.3%)	7 (70%)	41.2%	
					
Mass on USG	Yes	27 (77.1%)	9 (90%)	25%	0.370
	No	8 (22.9%)	1 (0%)	11.1%	
					
MIC USG	Yes	2 (5.7%)	3 (30%)	60%	***0.031***
	No	33 (94.3%)	7 (70%)	17.5%	
					
Mass + MIC on USG	Yes	1 (2.9%)	3 (30%)	75%	***0.008***
	No	34 (97.1%)	7 (70%)	17.1%	
					
Needle size	14 Gauge	30 (85.7%)	9 (90%)	23.1%	0.725
	11 Gauge	5 (14.3%)	1 (0%)	16.7%	
					
Number of Cores	≤ 5	30 (85.7%)	8 (80%)	21.1%	0.660
	> 5	5 (14.3%)	2 (20%)	28.6%	

MMG: mammogram, MIC: microcalcification, USG: ultrasonography

Univariate analysis revealed that age (≥ 50 years) at the time of core needle biopsy (p = 0.006), size on imaging (> 10 mm; p = 0.033), and combined mass with microcalcification on sonography (p = 0.029) were associated with underestimation (Table 2). When those three factors were included in multivariate analysis, only age at the time of core needle biopsy (p = 0.035, HR 6.201, 95% CI 1.135-33.891) was found to be an independent predictor of malignancy, whereas size on imaging and combined mass with microcalcification on sonography were negative predictors. (Table 3)

**Table 2:** Results of univariate analysis

	**HR**	**95% CI**	**P-value**
Age	9.333	1.911-45.583	***0.006***
Mass MMG	2.1	0.434-10.168	0.357
MIC MMG	2.875	0.579-14.275	0.196
Mass + MIC MMG	8.857	0.701-111.937	0.092
Lesion size	5.367	1.147-25.105	***0.033***
Mass USG	2.667	0.292-24.345	0.385
MIC USG	4.571	0.758-27.577	0.097
Mass + MIC USG	14.571	1.315-161.418	***0.029***
Needle size	1.50	0.155-14.557	0.727
Number of cores	1.50	0.244-9.219	0.662

HR: hazard ratio; CI: confidence interval; MMG: mammogram; MIC: microcalcification; USG: ultrasonography

**Table 3:** Results of Multivariate analysis.

	**HR**	**95% CI**	**P-value**
Age	6.201	1.135-33.891	0.035
Lesion size	2.878	0.474-17.465	0.250
Mass + MIC USG	4.571	0.288-72.609	0.281

HR: hazard ratio; CI: confidence interval; MMG: mammogram; MIC: microcalcification; USG: ultrasonography

## Discussion

In the present study, clinico-pathological and radiological findings of ADH diagnosed by CNB were assessed to clarify predictors that could be useful in distinguishing between ADH and cancer containing DCIS. ADH is a borderline lesion on histology that is difficult to distinguish from low grade DCIS on the small tissue sample provided by CNB. Because of this difficulty, clinico-pathologic and radiologic findings that can help discriminate between ADH and DCIS are valuable in planning patient management.

Although some variables like lesion size, combined mass, and microcalcification on sonography also tended to increased underestimation, only age at the time of biopsy (≥ 50 years) was presently determined to be an independent predictive factor for breast cancer at surgical excision in patients with diagnosed ADH at CNB. Consistent with our findings, Ko *et al *[18] observed an increase in underestimation rates in subjects aged 50 years and older, microcalcification on mammography and, lesion size > 15 mm.

Several studies have examined various mammographic, clinical, and pathological factors that may predict the presence of a more significant lesion on surgical excision after a CNB diagnosis of ADH [12,19-21]. It is believed that the variability of cancer rates depends on the size and features of the mammographic lesion, size of the biopsy needle, extent and completeness of sampling of the mammographic target lesion, histological criteria used to diagnose ADH versus DCIS and/or usual hyperplasia, and the threshold for surgical excision.

Use of vacuum assistance and more extensive sampling have improved the underestimation of carcinoma on surgical biopsy after a diagnosis of ADH on CNB from 33%-48% [2,21] to 7%-35% [14,22-25]. Although reduced underestimation with use of an 11-gauge vacuum-assisted device is explained by larger sample volumes, the number of specimens obtained presently appeared not to be correlated with a lower rate of underestimation. These results are similar to those of a previous study [12], in which the investigators found that specimen numbers per lesion did not correlate with underestimation, but that complete lesion removal did correlate with degree of underestimation. These findings indicate that targeting precision is more important than sample numbers. Further studies with more cases are needed to determine whether complete lesion removal at sonographically guided 11-gauge vacuum-assisted biopsy can reduce the rate of underestimation of ADH.

Jackman *et al*, [26] recognized that as the maximum diameter of the mammographic lesion increased, so does the rate of ADH underestimation. Also, in the present study, lesion size on imaging of > 1 cm increased underestimation rates. However, lesion size was not an independent predictor upon multivariate analysis.

Microcalcification with or without a mass has been reported to be the most common finding for both ADH (58% 88%) and DCIS (68% 98%) [8,27-29]. On histological examination, Helvie *et al *[10] found that the calcifications in mammary ducts within areas of ADH, without cell necrosis. In DCIS, the calcifications develop in secretions and are dystrophic calcifications secondary to necrotic tumor cells [30,31]. These histological differences could potentially be associated with different mammographic findings. But, those detailed variables were not addressed in the present study. Presently, only the combined sonographic finding of mass and microcalcifications was a significant predictive factor for underestimation in the univariate analysis. It has been suggested that microcalcification on mammography is an independent predictor of malignancy at follow-up surgical excision in patients diagnosed with ADH at CNB [18]. Our results did not reveal statistical significance in this regard.

Limitations of the present study include its retrospective nature and that it did not involve a randomized series of patients. Furthermore, 24 (34.8%) of the 69 ADH cases did not undergo surgical excision and were therefore excluded. It is possible that cases with a lower possibility of malignancy were recommended for imaging follow-up rather than surgical excision, which could affect the underestimation rate and other results.

## Conclusion

Only age at the time of biopsy (≥ 50 years) is an independent predictive factor for breast cancer at surgical excision in patients with diagnosed ADH at CNB. Identification of patients with ADH diagnosed by CNB who can be spared surgical excision is an area of active investigation. However, at present, clinical, radiologic, and pathologic factors on which to base this decision have not been consistently identified. So, for patients diagnosed with ADH at CNB, only complete surgical excision is a suitable treatment option because we could not find any combination of factors that can safely predict the absence of DCIS or invasive cancer in a case of ADH.

## Competing interests

The authors declare that they have no competing interests.

## Authors' contributions

BJC carried out the statistical analysis, participated in the sequence alignment and drafted and described the manuscript. AL carried out the Pathologic diagnosis. BJS and SSJ conceived of the study, and participated in its design and coordination and helped to draft the manuscript. All authors read and approved the final manuscript.
